# Smartphone application improves fertility treatment-related literacy in a large-scale virtual randomized controlled trial in Japan

**DOI:** 10.1038/s41746-021-00530-4

**Published:** 2021-11-30

**Authors:** Ryo Yokomizo, Akari Nakamura, Makoto Sato, Risa Nasu, Maaya Hine, Kevin Y. Urayama, Hiroshi Kishi, Haruhiko Sago, Aikou Okamoto, Akihiro Umezawa

**Affiliations:** 1 Center for Regenerative Medicine, National Center for Child Health and Development Research Institute, 2-10-1 Okura, Setagaya, Tokyo 157-8535 Japan; 2 Department of Obstetrics and Gynecology, The Jikei University School of Medicine, 3-25-8 Nishi-Shinbashi, Minato, Tokyo 105-8461 Japan; 3 Center for Maternal-Fetal, Neonatal and Reproductive Medicine, National Center for Child Health and Development, 2-10-1 Okura, Setagaya, Tokyo 157-8535 Japan; 4 Department of Healthcare Business, MTI Ltd., 3-20-2 Nishishinjuku, Shinjuku, Tokyo 163-1435 Japan; 5 Department of Social Medicine, National Center for Child Health and Development, 2-10-1 Okura, Setagaya, Tokyo 157-8535 Japan; 6 Graduate School of Public Health, St. Luke’s International University, 3-6-2 Tsukiji, Chuo-ku, Tokyo 104-0045 Japan

**Keywords:** Patient education, Medical research

## Abstract

People of reproductive age have unmet needs related to deficiencies in fertility literacy. Here, we aimed to investigate whether providing fertility-related information via a smartphone application could improve fertility treatment-related literacy in participants. We performed a randomized control-group pretest posttest study and recruited participants between June 18 and 25, 2020. Participants’ fertility treatment-related literacy was assessed with a pretest that comprised of 28 questions and participants were allocated with stratified randomization to either intervention group or control group. The intervention comprised a one-week smartphone application-based provision of information on fertility-related information and the control group received general information about women’s healthcare. Effectiveness of intervention was assessed using a posttest. A total of 4137 participants were administered the questionnaire and pretest, among which 3765 participants (91.0 %) responded and were randomly allocated into either the intervention group (*N* = 1883) or the control group (*N* = 1882). A significantly higher posttest mean score was observed for the intervention group compared to the control group (*P* = 0.0017). We also observed that posttest scores were significantly improved compared to pretest scores in both the intervention and control group (*P* < 0.001). When examining by specific test question, the proportion answering correctly increased at posttest compared to pretest for both intervention and control groups (*P* < 0.001). Furthermore, the intervention group showed a greater mean difference between posttest and pretest scores than the control group (*P* < 0.001). In conclusion, educational intervention using a smartphone application contributed to enhancing fertility treatment-related literacy.

## Introduction

Infertility is defined as a failure to conceive after 12 months of regular and unprotected sexual intercourse, and is estimated to affect 8–12% of couples of reproductive age worldwide^1^. With declining birth rates experienced by developed countries, infertility issues will add to economic hardship and is of significant public health importance. Internationally, it has been estimated that 56% of couples have sought medical care including fertility-related issues and treatment^2^, which is believed to be an underestimate of the overall population that experiences infertility challenges. In such contexts, Japan is recognized as one of the most active countries in reproductive medicine globally^3^, where nationwide surveys have shown that 1 in 5.5 couples of reproductive age had undergone fertility treatment (http://www.ipss.go.jp/site-ad/index_english/Survey-e.asp).

Treatments available for couples experiencing infertility issues include ovulation induction with timed intercourse, artificial insemination with husband (AIH), in vitro fertilization (IVF) and intracytoplasmic sperm injection^4^. According to the definition used by Centers for Disease Control and Prevention, assisted reproductive technology (ART) includes all fertility treatments in which either eggs or embryos are handled (https://www.cdc.gov/art/whatis.html). Artificial insemination is applied to achieve fertilization at the timing of ovulation, and this procedure is not included in ART. Alternatively, mature oocytes are retrieved directly from the ovary for fertilization in an IVF protocol. A typical IVF cycle includes gonadotropin stimulation, followed by oocyte retrieval. Oocytes can be fertilized in vitro either mixed with sperm or with intracytoplasmic sperm injection. Fertilized eggs (embryos) are cultured under optimal conditions, then transferred into the uterus (embryo transplantation). Reproductive endocrinologists suggest appropriate therapeutic strategies for the patients based on clinical examinations, such as hormonal level measurements, ultrasonographic images of the uterus and ovaries, radiological findings (hysterosalpingography), and sperm analysis. However, for patients, the interpretation of examination results may be overly complicated and complex, and patients may have difficulty making sense of their own fertility problems. Indeed, people are unaware of their own fertility potential, the constraints on their fertility, the signs, symptoms or preventable causes of fertility problems^5–7^, or the available ART that can shape their reproductive lives^7^.

Recently, the Internet has become a common source for fertility treatment-related information, and social media is viewed as a potentially effective avenue for dissemination of fertility-related news and education^8^. It has been reported that a median of 76% of the population among 18 advanced economies surveyed have smartphones^9^. In particular, considering the high rate of smartphone ownership in Japan (https://www.soumu.go.jp/johotsusintokei/whitepaper/ja/r01/html/nd232110.html), it is likely that couples of reproductive age would access information about fertility and increase their literacy using the Internet via smartphone. Indeed, in the field of cardiology, it has been demonstrated that digital patient education can increase patient literacy, and also improve the quality of life and lower feelings of depression and anxiety^10^. Online information is easily accessible; however, the information does not always reflect evidence-based recommendations^11^. In a previous study on the availability of Internet-based information for people trying to conceive, it was shown that inaccurate and non-evidence-based statements are frequently put forward in online websites which are readily accessible to people trying to conceive who may partake in online communities and distribute this information^12^. Although the evidence is still limited, considering the ease of access to low quality information, it is conceivable that misinformation and/or poor quality information may adversely affect patients seeking fertility treatment; thus, innovative approaches to providing both accessible and accurate information are desired.

Accumulating evidence indicates that providing quality-assured information could improve patients’ fertility-related knowledge and help decision-making^13–15^. Within the Japanese context, it is reasonable to hypothesize that providing high-quality information via a smartphone application can be an appealing strategy to help offset the damage incurred from vastly accessible low-quality information. To the best of our knowledge, the effectiveness of a smartphone application to improve literacy on fertility treatment has not been investigated. The smartphone application, *Luna Luna*, is the most popular female health care application in Japan and has been widely downloaded by about 16 million people. As one of its features, this application provides high-quality information about fertility treatment developed under the supervision of a reproductive endocrinology specialist.

In this study, using this *Luna Luna* platform to educate and collect large-scale data from users, we conducted a randomized control-group pretest posttest study to investigate whether providing high-quality fertility treatment information has the ability to enhance fertility treatment-related literacy among a large Japanese population. We also expected that the findings from this study may provide insight for other countries faced with similar challenges regarding reproductive health.

## Results

### Demographic characteristics of participants

**Fig. 1:**
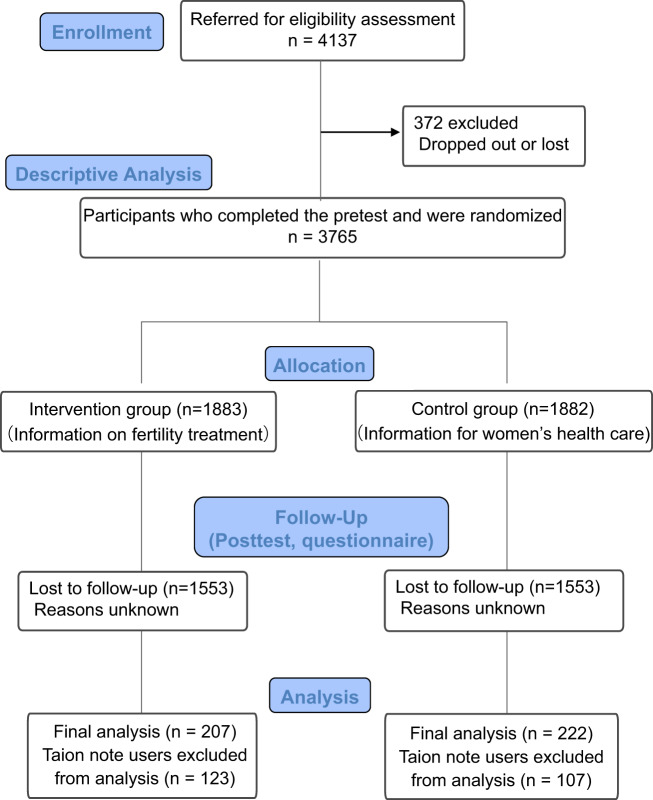
Flow chart of participant inclusion of the randomized control-group pretest posttest study (CONSORT Flow Diagram). A total of 4137 Luna Luna users agreed to participate and were administered the questionnaire and pretest, among which 3765 participants responded and were randomly allocated into either the intervention group (*N* = 1883) or the control group (*N* = 1882). After the follow-up, the posttest was completed by 659 participants and after excluding duplicate users of Luna Luna and Taion note, 207 participants in the intervention group and 222 participants in the control group were available for pretest-posttest analysis.

A total of 4137 *Luna Luna* users agreed to participate and were administered the questionnaire and pretest (Fig. 1), among which 3765 participants (91.0%) responded and were randomly allocated into either the intervention group (*N* = 1883) or the control group (*N* = 1882). Characteristics of participants appeared similar between the groups reflecting that the randomization was successful in producing a balance in baseline characteristics (Table 1). Notably, mean age (32.6 years), duration of desire for pregnancy (15.2 months), and the proportion receiving fertility treatment (15.6%) among the participants appeared similar to the characteristics of the general Japanese population (http://www.ipss.go.jp/ps-doukou/e/doukou15/Nfs15_gaiyoEng.html).

**Table 1:** Characteristics of participants who completed the pretest.

Parameter	*N* (%), mean ± SD	
	Control group	Intervention group
Participants	1882	1883
Sex		
Female	1872 (99.5)	1866 (99.1)
Male	10 (0.5)	15 (0.8)
Do not want to answer	0 (0.0)	2 (0.1)
Age (years)^a^	32.5 ± 5.0	32.6 ± 5.3
Education		
Middle high school/high school	445 (23.6)	434 (23.0)
Vocational school	396 (21.0)	380 (20.2)
National institute of technology/junior college	260 (13.8)	257 (13.6)
University/graduate school	746 (40.0)	785 (41.7)
Do not want to answer	35 (1.9)	27 (1.4)
Occupation		
Full-time job	1032 (54.8)	1035 (55.0)
Temporary job/Contract employee/Part-time job	455 (24.2)	430 (22.8)
Employer/Self-employment/Freelance	61 (3.2)	87 (4.6)
Not employed	295 (15.7)	272 (14.4)
Student	2 (0.1)	10 (0.5)
Others	24 (1.3)	36 (1.9)
Do not want to answer	13 (0.7)	13 (0.7)
Medical/healthcare background		
None	1401 (74.4)	1429 (75.9)
Medical professional	342 (18.2)	312 (16.6)
Worked at medical/healthcare company	102 (5.4)	99 (5.3)
Studied medicine/healthcare	17 (0.9)	16 (0.8)
Do not want to answer	20 (1.1)	27 (1.4)
Partner		
Yes	1828 (97.1)	1831 (97.2)
No	46 (2.4)	45 (2.4)
Do not want to answer	8 (0.4)	7 (0.4)
Lives with partner		
Yes	1638 (89.6)	1618 (88.4)
No	190 (10.4)	209 (11.1)
Do not want to answer	1 (0.1)	4 (0.2)
Annual household income		
<5 million JPY	555 (29.5)	516 (27.4)
5–10 million JPY	890 (47.3)	869 (46.1)
≥10 million JPY	200 (10.6)	233 (12.4)
Do not want to answer	237 (12.6)	265 (14.1)
Prior pregnancy		
Yes	728 (38.7)	681 (36.2)
No	1132 (60.1)	1181 (62.7)
Do not want to answer	22 (1.2)	21 (1.1)
Conception method^b^		
Natural	665	615
Timed intercourse	45	42
Artificial insemination	13	10
In vitro fertilization	20	21
Others	11	10
Do not want to answer	2	3
Prior delivery		
Yes	498 (68.3)	461 (67.7)
No	229 (31.4)	216 (31.7)
Do not want to answer	2 (0.3)	4 (0.6)
Desire for pregnancy		
Yes	1749 (92.9)	1756 (93.3)
No	111 (5.9)	105 (5.6)
Do not want to answer	22 (1.2)	22 (1.2)
Duration of desire for pregnancy (months)^c^	14.6 ± 19.7	15.7 ± 21.8
Action for pregnancy		
Natural (not intended)	713 (40.8)	702 (40.0)
Self-management without medical advice	482 (27.6)	484 (27.6)
Wondering about fertility treatment	155 (8.9)	188 (10.7)
Receiving fertility treatment	282 (16.1)	266 (15.1)
Cessation of fertility treatment	77 (4.4)	85 (4.8)
Others	38 (2.2)	23 (1.3)
Do not want to answer	2 (0.1)	8 (0.5)
Type of fertility treatment^b^		
Counseling	133	141
Screening test for infertility	198	189
Timed intercourse	240	242
Artificial insemination	74	68
In vitro fertilization	46	51
Others	5	6
Do not want to answer	3	2
Type of medical institution for fertility treatment		
Specialty clinics for fertility treatment	138 (38.4)	128 (36.5)
General gynecology clinics/hospital	196 (54.6)	202 (57.5)
General hospital/University hospital	20 (5.6)	19 (5.4)
Others	3 (0.4)	1 (0.1)
Do not want to answer	2 (0.3)	1 (0.1)
Fertility consultation for the partner		
Yes	149 (8.1)	135 (7.4)
No	1676 (91.7)	1688 (92.2)
Do not want to answer	3 (0.2)	8 (0.4)

*SD* standard deviation

^a^Participants aged 60 or older were assigned an age of 60 (1 participant in intervention group).

^b^Included only a subset of the participants for whom the question applied; more than one answer may have been selected.

^c^Participants whose duration of desire for pregnancy was over 10 years were assigned a value of 10 years (120 months) (33 in intervention group and 23 in control group).

### Fertility and treatment-related literacy in Japan

In the pretest, the overall mean test score was 57.8%, and scores were similar between the intervention and control groups (Table 2). Focusing on each question, there were no marked difference in the proportion answering correctly between the two groups. Interestingly, the topic of female age and pregnancy depicted in Question 6 was familiar to the majority participants (87.9%). In contrast, we observed that for 14 questions, less than half of participants answered them correctly. In particular, participants tended to incorrectly answer questions related to the proportion of couples with infertility problems in Japan (Question 12: controls- 47.8%; intervention- 48.8%), examinations performed in general fertility treatment (Question 18–21), and procedures for artificial insemination (Question 24: controls- 35.9%; intervention- 36.6%). Topics related to clinical practice in fertility treatment (Questions 18–21) appeared to be poorly understood in general. In addition, participants’ perception of pregnancy success rates for artificial insemination and assisted reproductive technology was higher than the actual observed rates (Question 25: controls- 20.9%; intervention- 21.3%, Question 28: controls- 31.8%; intervention- 31.0%).

**Table 2:** Pretest scores and numbers answering correctly by question.

Question number	Topics of question	Control group *N* = 1882	Intervention group *N* = 1883	*P* value
1	Physiology	1714 (91.1)	1712 (90.9)	0.913
2	Physiology	920 (48.9)	934 (49.6)	0.684
3	Physiology	1541 (81.9)	1524 (80.9)	0.481
4	Physiology	1548 (82.3)	1540 (81.8)	0.740
5	Physiology	1367 (72.6)	1400 (74.3)	0.248
6	Physiology	1655 (87.9)	1656 (87.9)	1.000
7	Clinical	662 (35.2)	643 (34.1)	0.530
8	Clinical	1859 (98.8)	1857 (98.6)	0.775
9	Clinical	1648 (87.6)	1639 (87.0)	0.664
10	Lifestyle	1418 (75.3)	1472 (78.2)	0.044
11	Lifestyle	944 (50.2)	890 (47.3)	0.081
12	General	900 (47.8)	918 (48.8)	0.590
13	General	1514 (80.4)	1527 (81.1)	0.644
14	Male involvement	1263 (67.1)	1263 (67.1)	1.000
15	Male involvement	1573 (83.6)	1539 (81.7)	0.145
16	Financial	1013 (53.8)	963 (51.1)	0.106
17	Financial	1301 (69.1)	1350 (71.7)	0.091
18	Test and treatment	419 (22.3)	407 (21.6)	0.659
19	Test and treatment	796 (42.3)	758 (40.3)	0.216
20	Test and treatment	699 (37.1)	698 (37.1)	0.990
21	Test and treatment	529 (28.1)	548 (29.1)	0.523
22	Test and treatment	1604 (85.2)	1608 (85.4)	0.921
23	Test and treatment	787 (41.8)	798 (42.4)	0.752
24	Test and treatment	675 (35.9)	689 (36.6)	0.668
25	Test and treatment	393 (20.9)	402 (21.3)	0.756
26	Test and treatment	776 (41.2)	769 (40.8)	0.832
27	Test and treatment	314 (16.7)	357 (19.0)	0.075
28	Test and treatment	598 (31.8)	584 (31.0)	0.640
Overall test score (mean ± SD)		57.8 ± 16.9	57.7 ± 16.9	0.986

*SD* standard deviation

### Smartphone application enhanced fertility treatment-related literacy

The posttest was completed by 659 participants (17.5%) and after excluding duplicate users of *Luna Luna* and *Taion note*, 207 participants in the intervention group and 222 participants in the control group were available for pretest-posttest analysis (Fig. 1). Demographic characteristics of these participants appeared similar between the intervention and control groups (Supplementary Table 1). Participants represented a broad range of educational backgrounds and reflected the distribution observed in the overall general population. However, in comparing the demographic characteristics of participants who did and did not complete the posttest, there were significant differences between the two groups in age, overall pretest score, proportion living with a partner, and action for pregnancy suggesting that those who completed this trial may have had greater motivation and diligence to learning about fertility (Supplementary Table 2).

Examination of the overall posttest performance showed that the number of questions for which less than 50% of participants answered correctly decreased to 4 questions in the posttest (Question 18, 25, 27, 28) among the intervention group (Table 3). These four questions were related to details of fertility treatment and were expected to be challenging concepts to understand through a smartphone application.

**Table 3:** Posttest scores and numbers answering correctly by question among participants completing the posttest.

Questions	Topics of question	Control group (*N* = 222), *N* (%)		*P* value	Intervention group (*N* = 207), *N* (%)		*P* value
		Pretest	Posttest		Pretest	Posttest	
1	Physiology	203 (91.4)	213 (95.9)	NS	189 (91.3)	193 (93.2)	NS
2	Physiology	128 (57.7)	143 (64.4)	0.044	119 (57.5)	153 (73.9)	<0.001
3	Physiology	182 (82.0)	192 (86.5)	NS	170 (82,1)	193 (93.2)	<0.001
4	Physiology	191 (86.0)	186 (83.8)	NS	172 (83.1)	191 (92.3)	<0.001
5	Physiology	173 (77.9)	180 (81.1)	NS	169 (81.6)	172 (83.1)	NS
6	Physiology	204 (91.9)	207 (93.2)	NS	192 (92.8)	192 (92.8)	NS
7	Clinical	85 (38.3)	101 (45.5)	0.033	58 (28.0)	128 (61.8)	<0.001
8	Clinical	221 (99.5)	221 (99.5)	NS	205 (99.0)	201 (97.1)	NS
9	Clinical	202 (91.0)	209 (94.1)	0.039	186 (89.9)	196 (94.7)	0.013
10	Lifestyle	179 (80.6)	185 (83.3)	NS	172 (83.1)	179 (86.5)	NS
11	Lifestyle	123 (55.4)	128 (57.7)	NS	119 (57.5)	141 (68.1)	0.005
12	General	113 (50.9)	109 (49.1)	NS	103 (49.8)	121 (58.5)	NS
13	General	192 (86.5)	194 (87.4)	NS	176 (85.0)	184 (88.9)	NS
14	Male involvement	138 (62.2)	149 (67.1)	NS	138 (66.7)	141 (68.1)	NS
15	Male involvement	176 (79.3)	173 (77.9)	NS	161 (77.8)	175 (84.5)	0.024
16	Financial	117 (52.7)	145 (65.3)	<0.001	102 (49.3)	135 (65.2)	<0.001
17	Financial	172 (77.5)	184 (82.9)	NS	160 (77.3)	177 (85.5)	0.002
18	Test and treatment	52 (23.4)	74 (33.3)	0.002	35 (16.9)	95 (45.9)	<0.001
19	Test and treatment	107 (48.2)	116 (51.7)	NS	92 (44.4)	107 (51.7)	0.032
20	Test and treatment	86 (38.7)	100 (45.0)	NS	77 (37.2)	111 (53.6)	<0.001
21	Test and treatment	74 (33.3)	101 (45.5)	<0.001	64 (30.9)	107 (51.7)	<0.001
22	Test and treatment	194 (87.4)	198 (89.2)	NS	185 (89.4)	196 (94.7)	0.007
23	Test and treatment	103 (46.4)	126 (56.8)	0.002	91 (44.0)	133 (64.3)	<0.001
24	Test and treatment	92 (41.4)	87 (39.2)	NS	95 (45.9)	106 (51.2)	NS
25	Test and treatment	45 (20.3)	49 (22.1)	NS	37 (17.9)	64 (30.9)	<0.001
26	Test and treatment	111 (50.0)	134 (60.4)	0.001	106 (51.2)	134 (64.7)	<0.001
27	Test and treatment	32 (14.4)	42 (18.9)	NS	34 (16.4)	62 (30.0)	<0.001
28	Test and treatment	73 (32.9)	79 (35.6)	NS	62 (30.0)	89 (43.0)	0.002
Overall test score (mean ± SD)		60.6 ± 17.5	64.8 ± 17.7	<0.001	59.9 ± 17.4	70.3 ± 18.3	<0.001

*NS* not significant, *SD* standard deviation

**Fig. 2:**
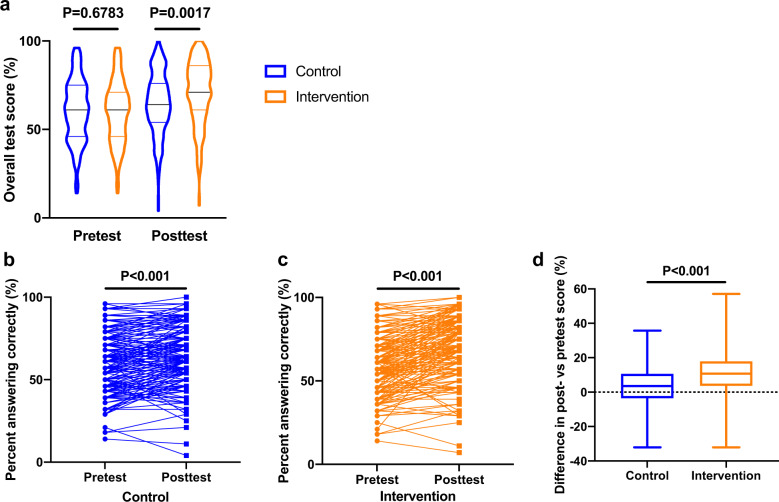
Effectiveness of intervention. **a** overall test score for pre- and posttest in control and intervention group. Violin plots show the distribution of overall test score. Significantly higher posttest mean scores were observed for the intervention group (*N* = 207) compared to the control group (*N* = 222) (*P* = 0.0017, Mann–Whitney U test). Center line, median; upper and lower lines, upper and lower quartiles. Percent answering correctly in (**b**) control group (28 questions) and (**c**) intervention group (28 questions). Line graphs show that the proportion answering correctly appeared to be generally increased at posttest compared to pretest for the control groups (*P* < 0.001) and the intervention group (*P* < 0.001) using the McNemar test. **d** Difference in posttest versus pretest scores in control and intervention group. Box plots show the difference in posttest versus pretest scores, and on average, greater improvements in the intervention group (*N* = 207) than the control group (*N* = 222) were observed (*P* < 0.001, Wilcoxon matched-pairs signed rank test). Center line, median; box limits, upper and lower quartiles.

Next, we analyzed the effectiveness of the intervention in this study (Fig. 2). For the pretest, the overall mean test scores were similar between the control and intervention groups as a result of the randomization, but significantly higher posttest mean scores were observed for the intervention group compared to the control group (*P* = 0.0017) (Fig. 2a). Interestingly, we also observed that posttest scores were significantly improved compared to pretest scores in both the intervention group and control group (*P* < 0.001) (Table 3). When examining by specific test question, the proportion answering correctly appeared to be generally increased at posttest compared to pretest for the intervention (*P* < 0.001) and control (*P* < 0.001) groups (Table 3, Fig. 2b and c). There was significant improvement in questions that covered topics related to “Physiology” and “Test and treatment”, and notably, over 30% improvement for Question 7 (clinical significance of anti-Müllerian hormone (AMH)) in the intervention group. Among the questions which had less than 50% answering correctly in the pretest, more questions had improved response rates at posttest in the intervention group (10 of 12 questions) compared to the control group (4 of 10 questions). Furthermore, comparing the difference in posttest versus pretest scores between the two groups showed, on average, greater improvements in the intervention group than the control group (*P* < 0.001) (Fig. 2d). These findings suggest that provision of fertility treatment-related information via the smartphone application may contributed to enhancement of fertility literacy.

**Fig. 3:**
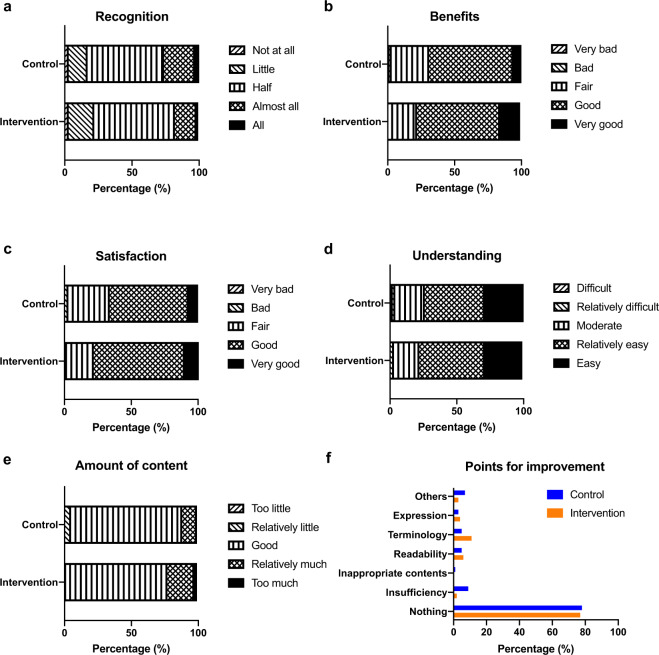
Responses to the questionnaire assessing the quality of the smartphone application and the provided information. Bar charts show the distribution of the assessment by the participants regarding (**a**) Recognition, (**b**) Benefits, (**c**) Satisfaction, (**d**) Understanding, (**e**) Amount of content, and (**f**) Points for improvement for the smartphone application and the provided information. The contents provided were considered adequate and highly satisfactory for most of the participants.

**Fig. 4:**
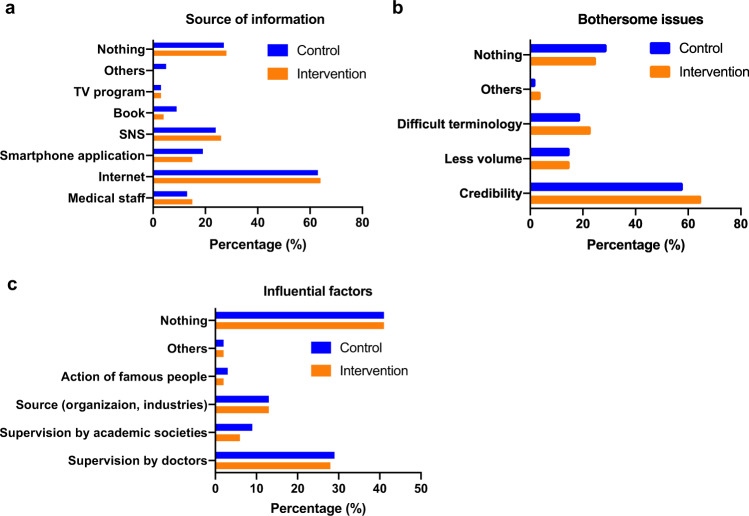
Usual habits for gathering fertility-related information. Bar charts show the responses from the participants regarding (**a**) source of information, (**b**) bothersome issues, and (c) influential factors when gathering fertility-related information. Most of the participants utilized Internet-related tools and they were bothered about potential credibility issues of the information; however, most indicated “none” when asked about the influence of source providing the information.

We further examined the quality of the smartphone application based on information provided in the follow-up questionnaire (Fig. 3). We asked the participants about Recognition of content (Fig. 3a), Benefits of the content (Fig. 3b), Satisfaction (Fig. 3c), Ease of understanding (Fig. 3d), Amount of content (Fig. 3e), and Points for improvement (Fig. 3f). Based on the responses, we found that the contents we provided were considered adequate and highly satisfactory for most of the participants. Furthermore, in the questionnaire, we investigated the usual habits of the participants for gathering fertility-related information and asked about “Source of information” (Fig. 4a), “Bothersome issues” (Fig. 4b), and “Influential factors” (Fig. 4c). We identified that most of the participants utilized Internet-related tools, and they were bothered about potential credibility issues of the information; however, most indicated “none” when asked about the influence of source providing the information. These characteristics may be important to consider when developing a more efficient and high-quality tool.

## Discussion

As a primary finding of this study, we found that fertility- and treatment-related information provided via a smartphone application contributed to enhancing fertility literacy. In this study, we developed a fertility literacy assessment tool based on the CFKS-J, which has been used in international comparative studies across 79 countries, including Japan, and its validity and reliability is well-recognized^13,16^. However, CFKS-J does not cover fertility treatment-related literacy; thus, we conducted this study with an emphasis on the effectiveness of an educational intervention on fertility treatment-related literacy. This is the largest randomized control trial to examine fertility treatment-related literacy using a smartphone application in a general population, including those who have already received fertility treatment. Questions included in this current study are those which we expect can be answered by people who have already received fertility treatment. In other words, if a couple has a chance to receive treatment due to infertility, it is hopeful that this level of understanding is acquired. Even if not currently undergoing fertility treatment, it would be worthwhile for the general public to know this information in case infertility becomes an issue for them in the future. Furthermore, it is also valuable for medical professionals to know the extent to which these medical issues are understandable for the general public. Patient-centered care, good communication and a strong patient–healthcare provider relationship are essential for effective management of patients with infertility^17^. These measures may also improve patient adherence to treatment schedules, reduce the physical and emotional burdens associated with treatment, and decrease the rates of treatment discontinuation^18,19^. Finally, by improving fertility treatment literacy in general, including the degree of effectiveness for procedures like ART, it might help to enhance self-determination regarding pregnancy issues^17^.

Interestingly, significant improvement of literacy in the control group was observed in this study. Similar to the researches using smartphone application^20,21^, it is possible that the control group experienced something similar to a placebo effect. Although the content provided to the controls did not specifically cover infertility issues, it is possible that the act of studying women’s health issues may have helped them to recall topics they have previously heard about or studied. We also cannot rule out the possibility that the controls may have accessed other education materials prior to the posttest as described in the limitations. In addition to assessing the effectiveness of the education intervention, important insights were gained from observing the pattern of incorrect answers and the characteristics associated with them. First, there appeared to be high expectations for fertility treatment. Participants’ perception of pregnancy success rates for AIH and ART was higher than the actual observed rates. A previous study also showed that large proportions of men and women from the general population have limited knowledge of the factors influencing fertility, and tended to overestimate the ability of medically assisted reproduction to overcome age-related infertility^22^. Second, most participants misunderstood the procedures for AIH as IVF, suggesting that Japanese people do not have sufficient knowledge about types of fertility treatment. Third, participants believe that if they are examined with a certain fertility test, everything can be uncovered, i.e., they do not understand what type of information each test reveals. In decision-making, accurate understanding of what people desire and what people understand should be critical.

With growing fertility issues experienced by many countries, the findings from this Japanese context may help to inform strategies for addressing similar circumstances in other regions of the world as well. We assumed that people with concerns on credibility would be influenced by the source of the information; however, we observed that participants seem to obtain information without particularly caring about the source of information, even though they may have doubts about its reliability. This discrepancy may reflect information literacy circumstance of the Japanese population (https://www.soumu.go.jp/johotsusintokei/whitepaper/ja/h26/html/nc143120.html).

In Japan, care offered by reproductive endocrinologists for fertility management, including timed intercourse with ovarian stimulation, is covered by public insurance; however, medically-assisted reproduction, including AIH and ART, is not covered in principle and financial support is often needed (https://www.mhlw.go.jp/shingi/2006/10/s1018-7h01.html). Our study showed that people of childbearing age have a reasonable understanding of the public insurance system. In light of the Japanese government’s recent consideration of expanding public insurance to cover medically-assisted reproduction, the findings from our study that targeted the general population of Japan, provides important insight into the current population circumstance associated with fertility treatment (https://www8.cao.go.jp/shoushi/shoushika/law/pdf/r020529/shoushika_taikou_b1.pdf). In particular, in the context of understanding that fertility declines with age, we found that the participants are generally familiar with the age-related fertility decline, and this type of educational provision could enhance knowledge on the clinical significance of AMH which is recognized as a biomarker for ovarian reserve^23^. The Japanese government is considering to limit the application of public insurance support and subsidies based on the women’s age with reference to the success rate of fertility treatment (https://www.tokyo-np.co.jp/article/122691). In order to determine whether this policy would be generally accepted by the public, it would be worthwhile to gauge the current level of public understanding regarding age and fertility treatment success rates. These findings can contribute to evidence-based policy making when setting the age limit in applying for public insurance.

Providing information through a smartphone application may be considered acceptable since retrieving information through a smartphone application is in line with a modern day lifestyle, particularly in developed countries^9^. Indeed, our study found that most participants utilize Internet-based resources and tools, including a smartphone application, to obtain needed information, and there appeared to be a high level of satisfaction in the contents provided through this current study. In addition, compared to leaflet or textbook-based information, a smartphone application has potential to offer information in a variety of ways. For example, in a previous study, providing tailored oral and written information had a positive effect on participants’ knowledge of reproductive health^24^. In another study, chatbot was found to be a more effective tool rather than standard provision of information^25^. In addition to these methods, a smartphone application may offer alternatives such as movie-based learning and others.

Shortcomings of the smartphone application were also identified. As shown in this study, despite the high level of satisfaction, the degree of effectiveness of the intervention was lower than expected. It is possible that the participants reviewed the contents only briefly and may not have been able to fully absorb the information. According to a systematic review for the educational benefit of online-education, high satisfaction and equivocal or increased preference were observed due to ease of access; on the other hand, acknowledged disadvantages included lack of discussion intensity^26^. Accuracy, credibility, and insufficient absorption of the information are regarded as disadvantages of web-based education, and development of further sophisticated strategies may be warranted^11,12^. Furthermore, the media perpetuates misinformation by highlighting individual cases of conception in later years and suggesting that ART, such as IVF, can compensate fully for age-related decline in fertility^27^. Poor fertility knowledge may be a contributory factor to many people not achieving their goal of parenthood^28^. Results of this exploratory trial suggests that additional effort in delivery approach of the intervention may have the potential to increase the effectiveness.

While a smartphone application approach offered advantages in targeting a broad population and pursing a unique question, this study had certain limitations to acknowledge. First, as the intervention was educational material, it was not possible to blind participants to intervention group assignment. Knowledge of group assignment may have influenced the degree of effort placed on test performance. However, consistency in results between intervention group comparison, as well as pretest-posttest comparisons within each group supports the effectiveness of the smartphone application educational intervention. Second, we were not able to monitor the participants during the one-week educational provision and when completing the tests; thus, whether they accessed other resources and the possibility that participants looked for information in the time of posttest could not be addressed. However, as part of the study protocol, we requested participants to refrain from accessing other educational materials during the study period. In addition, smartphone application-based approaches to participant recruitment and intervention faces challenges with maintaining high follow-up rates. Without face-to-face contact or instilling a heightened sense of commitment to the study, we experienced high withdrawal rates, which were accounted for in our sample size calculations, but some analyses may have lacked statistical power to detect smaller than expected differences. This exploratory study may provide important insight to future studies using similar approaches. Third, the participants may have forgotten the contents by the time of the posttest. However, this was carefully considered when preparing the contents and method of providing information; as a result, for example, we adjusted the amount of information per day and set up the pages so that participants could refer to it later. Providing all information on one day and assessment of the effectiveness of intervention on the same day may have been possible, but this learning strategy may be too burdensome and not suitable for daily use. Furthermore, previous research similarly demonstrated that content related to fertility treatment details may be challenging concepts to understand through a smartphone application for a general population, particularly when the participants do not have previous experience with ART procedures^29^. If a bidirectional education style is provided, such as through a chatbot^25^, it may contribute to literacy improvement more effectively. Fourth, issues of generalizability should be acknowledged. While the large pretest baseline population allowed us to uniquely assess the current knowledge based of the general Japanese population, the external validity of the effects of the intervention should be interpreted with care. This virtual trial implemented through a smartphone application offered several advantages, but also contributed to a significant number participants not completing the trial to posttest. Focusing on the demographic characteristics of participants who did and did not complete the posttest, differences were observed for age and action for pregnancy. Consistent with our observations, previous studies have shown older participants to have higher fertility awareness than younger participants^6,14^. Also, research performed in Hungary showed that parents and those with ART experience were more knowledgeable than people without children and those without ART experience^29^. Thus, the intervention effects observed in this study may predominately apply to a population with heightened interest and motivation for learning about fertility-related issues. In addition, smartphone application use for this type of purpose may be skewed towards females who were self-motivated and of certain sociodemographic characteristics. Regarding the effect of sociodemographic variables, previous studies have reported a gender gap in which women had more knowledge about general fertility and ART than men^6,30–32^. As described, men make up half of the cause of infertility, and it is desirable to have an effective tool for providing information to men as well. In fact, our study showed that male partners rarely examined their own fertility. At baseline, a small number of male partners were recruited and enrolled in this study, but most did not complete the trial to posttest. It is possible that men still perceive reproductive issues to be primarily women’s domain which might have affected the response in this trial^33^. Additional studies that strategically target men may be needed for achieving better-quality interventions for fertility issues. As a study conducted in Japan, the current results may not be directly generalizable to populations in other countries where educational status and social support systems for fertility treatment may be different. However, infertility issues are prevalent worldwide, especially in developed countries, and this study conducted in Japan may provide insight into efforts in other countries as well.

In conclusion, this study demonstrated that educational intervention using a smartphone application platform may contribute to enhancing fertility treatment-related literacy. To further improve the effectiveness, future research may build on insights gained from this exploratory trial to develop more sophisticated educational strategies that benefit broad populations using smartphone-based approaches. In addition, our final goal should be to support couples with fertility issues, not only to increase their knowledge on fertility. The current study did not follow-up participants to assess their behavior changes, but we are planning additional investigation to see whether or not improved literacy maintains over time and leads to changes in habit and better outcomes (e.g., better decision-making, fewer adverse effects in fertility treatment, etc). Furthermore, long-term public health strategies are essential. In the UK, a group of stakeholders have set up the Fertility Education Initiative to increase opportunities for fertility education and to help ensure all women and men are able to make informed choices^34^. An example from Australia is “Your Fertility”, a fertility health promotion program funded by the government to improve awareness of modifiable factors that affect fertility and reproductive outcomes^22^. A similar type of system in Japan would contribute significantly to the well-being of couples and effect population health for the long-term.

## Methods

### Ethical statement

Ethical approval for the implementation of the present study was obtained from the Institutional Review Board of the National Center for Child Health and Development of Japan (approval number: 2019-184). All participants in the study agreed to be enrolled and selected the agreement button in *Luna Luna*, which equates to signing the informed consent form before inclusion. This randomized controlled trial was registered on 15 June 2020 with UMIN Clinical Trials Registry number UMIN000040721 (https://upload.umin.ac.jp/cgi-bin/ctr/ctr_view_reg.cgi?recptno=R000046393).

### Study design and population

We conducted a randomized control-group pretest posttest study among current application users of MTI Ltd.’s (http://www.mti.co.jp/eng/) *Luna Luna*. *Luna Luna* is a smartphone application that assists users in predicting their menstrual cycle and ovulation under normal physical conditions. The basic functions of this application are freely available on the Android and iOS platforms. This application is used for both pregnancy and contraception planning and for general health management, thus targeting the Japanese female population in general^35,36^.

Users were requested to participate in this study by sending in-application notifications about the outline of the research plan and how data collected from the application would be used. Furthermore, MTI Ltd. published a press release about the study, together with information on how to voluntarily participate. We recruited participants between June 18 and 25, 2020. The recruitment ended on June 25 because the number of participants reached to the pre-estimated number. At that time, the number of user who accessed the pages which requested participation for this study was 17,931. This number includes users who had simply glanced or unintentionally accessed the pages. We included users of *Luna Luna* who were over 20 years of age and excluded those who used both *Luna Luna* and MTI Ltd.’s *Taion note* application since fertility treatment-related information provided in this study can also be accessed by using the *Taion note* application.

### Procedures at baseline and pretest

We performed a study using the randomized control-group pretest posttest design in accordance with the CONSORT Flow Diagram (Fig. 1). We collected baseline information including participants’ gender, age, education level, occupation, annual household income, and number of pregnancies. We requested participants to complete a pretest consisting of 28 items regarding reproductive health derived from previously published research^16^ and additional items related to fertility treatment. The pretest was administered in Japanese, and a translated summary of topics in English is shown in Table 4 (full version and answers are shown in Supplementary Table 3). The pretest was developed based on the Japanese version of a well-known validated fertility literacy assessment tool called the Cardiff Fertility Knowledge Scale (CFKS-J)^16^, and questions covered the following topics: the physiology of pregnancy, the fertile window within the menstrual cycle, infertility risk factors, fertility treatment, and fertility treatment-related financial systems in Japan. Over a one-week period, participants were asked to complete the pretest by selecting from among three possible answers, or “I don’t know”. Participants were requested not to access other resources when completing the test. To ensure that the pretest was understandable to a general population and captured the intended content, we piloted the survey on a group of individuals at the National Center for Child Health and Development, and the final version was confirmed by clinical experts.

**Table 4:** Summary of contents included in the test for fertility literacy assessment by question.

Question	Contents
Question 1	Concept of fertilization
Question 2	Location of fertilization
Question 3	Lifespan of an oocyte
Question 4	Lifespan of a sperm
Question 5	Location of implantation
Question 6	Relationship between female age and pregnancy
Question 7	Clinical significance of AMH (anti-Müllerian hormone)
Question 8	Optimal time during menstrual cycle for pregnancy
Question 9	Proper location for measuring basal body temperature
Question 10	Effects of tobacco on fertility
Question 11	Relationship between lifestyle and fertility
Question 12	Proportion of couples with infertility problems in Japan
Question 13	Definition of infertility
Question 14	Men’s involvement in infertility
Question 15	Etiology of male infertility
Question 16	Public insurance for fertility treatment
Question 17	How to apply for the public financial subsidy for fertility treatment
Question 18	Relationship between blood test and ovarian function
Question 19	Availability of ultrasonography
Question 20	Availability of hysterosalpingography
Question 21	Availability of Huhner test
Question 22	How to perform a semen analysis
Question 23	Clinical practice pertaining to the timed intercourse
Question 24	Procedures for artificial insemination
Question 25	Pregnancy rate for artificial insemination
Question 26	Treatment flow for in vitro fertilization
Question 27	Culture duration after in vitro fertilization
Question 28	Pregnancy rate for in vitro fertilization

### Intervention and randomization

The intervention for this study comprised the administration of a week-long series of health-related information via the smartphone application *Luna Luna* which differed based on group assignment. The educational material was provided in a text-based format including images to supplement the explanations. In the intervention group, participants were provided with fertility- and treatment-related information, which were developed under the supervision of a reproductive endocrinology specialist (summarized in Supplementary Table 4). The information provided to the intervention group covered the topics that were included in the pretest. For the control group, participants were provided with general information about women’s health care and gynecologic disorders, which were developed under the supervision of an obstetrics and gynecology specialist (summarized in Supplementary Table 4). The content provided to the control group was carefully reviewed and it was confirmed that there was no direct topic overlap between the intervention groups.

We performed computer-based stratified randomization which involved separating the participants into 28 strata based on the performance on the pretest (28 total questions) in order to allocate them into two groups with similar distributions of high to low performance. Participants within each strata were assigned a unique randomly generated number, and were sequentially allocated to intervention or control group in an alternating fashion. Immediately after random allocation, participants were sent a notification, through the *Luna Luna* application, with instructions for accessing the educational material corresponding to their allocation.

### Posttest and follow-up questionnaire

On the last day of providing information, the posttest (same contents as the pretest) was administered under the same conditions as the pretest to measure the effectiveness of the intervention. The participants were given a week to answer the posttest. The pretest and posttest were scored separately by summing the number of correct answers divided by the total number of questions and multiplied by 100 (expressed as %), and the difference in the scores were also calculated.

Through administration of a follow-up questionnaire during the week subsequent to the posttest, we also assessed the participants’ satisfaction with the application and information provided, and their usual information-seeking behaviors about fertility treatment. We asked the participants the following questions regarding the application: “How much did you know about the contents (Recognition)?”, “How beneficial did you find it (Benefits)?”, “How satisfied were you with the content (Satisfaction)?”, “Was the content easy to understand (Understanding)?”, “How was the amount of content (Amount of content)?”, and “Were there any points to be improved (Points for improvement)?”. Regarding information-seeking behaviors for fertility-related information, we asked about “Source of information”, “Bothersome issues”, and “Influential factors”. Finally, at the end of the study, all participants were provided with the answers to the test questions, and further explanation was provided about the pretest and posttest as a way to provide them knowledge about fertility treatment. All study implementation procedures were pursued with close coordination between *Luna Luna* operators at MTI Ltd. and investigators at the National Center for Child Health and Development Research Institute.

### Sample size

Sample size calculations were performed based on detecting a difference of 20% in score between two groups and setting the type I and II error rate at 0.05 and 0.1, respectively. Mean score of in the control group was expected to be about 50%, resulting in a total number of 250 individuals (at a 1:1 scheme resulting in 125 per group). Considering an expectedly high withdrawal rate at follow-up for this application-based study, we empirically assumed that a baseline population of nearly 4000 participants allocated to each group would result in sufficient numbers based on the response and withdrawal rates experienced with previous surveys conducted through the *Luna Luna* application.

### Statistical analysis

Characteristics between the intervention group and the control group were compared using the Pearson’s chi-square test and Fisher’s exact test for categorical variables. Age and test score (on a 0–100% scale) were examined using the Mann–Whitney U test. Changes in test score between the pretest and posttest were compared using the Wilcoxon matched-pairs signed rank test. Effectiveness of intervention was analyzed by comparing the changes in test score and proportion answering correctly between the pretest and posttest within the intervention group and control group using the McNemar test. SPSS (IBM) and Prism 8.01 software (GraphPad, Inc.) were used for the statistical analyses. A two-sided *p* value of less than 0.05 was considered to be statistically significant for all analyses.

### Reporting summary

Further information on research design is available in the Nature Research Reporting Summary linked to this article.

## Supplementary information

Supplementary Information

Reporting Summary

## Data Availability

The datasets generated during and/or analyzed during the current study are available from the corresponding author on reasonable request.
