# Supplementary material for: Smartphone application improves fertility treatment-related literacy in a large-scale virtual randomized controlled trial in Japan
Source: NPJ Digit Med. 2021 Nov 30;4:163. doi: 10.1038/s41746-021-00530-4 (PMC8632894; doi:10.1038/s41746-021-00530-4)
Supplement: Supplementary file 1 — Supplementary Information [file 41746_2021_530_MOESM1_ESM.pdf]

Supplementary Table 1. Characteristics of participants who were included in the final posttest analysis

Parameters	N (%), mean ± SD			P-value
	Total	Control group	Intervention group	
Participants	429	222 (51.7)	207 (48.3)	
Sex				NS
Female	426 (99.3)	220 (99.1)	206 (99.5)	
Male	3 (0.7)	2 (0.9)	1 (0.5)	
Age (years)	33.5 ± 4.7	33.6 ± 4.5	33.5 ± 5.0	NS
Education				NS
Middle high school/high school	90 (21.0)	51 (23.0)	39 (18.8)	
Vocational school	91 (21.2)	44 (19.8)	47 (22.7)	
National institute of technology/junior college	57 (13.3)	34 (15.3)	23 (11.1)	
University/graduate school	186 (43.4)	92 (41.4)	94 (45.4)	
Do not want to answer	5 (1.2)	1 (0.5)	4 (1.9)	
Occupation				NS
Full-time job	225 (52.4)	114 (51.4)	111 (53.6)	
Temporary job/Contract employee/Part-time job	96 (22.4)	51 (23.0)	45 (21.7)	
Employer/Self-employment/Freelance	13 (3.0)	6 (2.7)	7 (3.4)	
Not employed	84 (19.6)	46 (20.7)	38 (18.4)	
Student	3 (0.7)	1 (0.5)	2 (1.0)	
Others	5 (1.2)	3 (1.4)	2 (1.0)	
Do not want to answer	3 (0.7)	1 (0.5)	2 (1.0)	
Medical/healthcare background				NS
None	319 (74.4)	167 (75.2)	152 (73.4)	
Medical professional	68 (15.9)	34 (15.3)	34 (16.4)	
Worked at medical/healthcare company	30 (7.0)	14 (6.3)	16 (7.7)	
Studied medicine/healthcare	6 (1.4)	5 (2.3)	1 (0.5)	
Do not want to answer	6 (1.4)	2 (0.9)	4 (1.9)	
Partner				NS
Yes	414 (96.5)	213 (95.9)	201 (97.1)	
No	13 (3.0)	8 (3.6)	5 (2.4)	
Do not want to answer	2 (0.5)	1 (0.5)	1 (0.5)	
Lives with partner				NS
Yes	381 (92.0)	194 (91.1)	187 (93.0)	
No	33 (8.0)	19 (8.9)	14 (7.0)	
Annual household income				NS
<5 million JPY	121 (28.2)	68 (30.6)	53 (25.6)	
5-10 million JPY	196 (45.7)	110 (49.5)	86 (41.5)	
≥10 million JPY	49 (11.4)	21 (9.5)	28 (13.5)	
Do not want to answer	63 (14.7)	23 (10.4)	40 (19.3)	
Prior pregnancy				NS
Yes	145 (33.8)	79 (35.6)	66 (31.9)	
No	280 (65.3)	140 (63.1)	140 (67.6)	
Do not want to answer	4 (0.9)	3 (1.4)	1 (0.5)	
Conception method[1]				NS
Natural	132	72	60	
Timed intercourse	12	6	6	
Artificial insemination	1	0	1	
In vitro fertilization	3	3	0	
Others	4	3	1	
Prior delivery				NS
Yes	104 (71.7)	56 (70.9)	48 (72.7)	
No	40 (27.6)	23 (29.1)	17 (25.8)	
Do not want to answer	1 (0.7)	0 (0.0)	1 (1.5)	
Desire for pregnancy				NS
Yes	406 (94.6)	207 (93.2)	199 (96.1)	
No	17 (4.0)	11 (5.0)	6 (2.9)	
Do not want to answer	6 (1.4)	4 (1.8)	2 (1.0)	
Duration of desire for pregnancy (months)[2]	14.3 ± 16.9	14.0 ± 15.7	14.6 ± 18.1	NS
Action for pregnancy				NS
Natural (not intended)	130 (32.0)	65 (31.4)	65 (32.7)	
Self-management without medical advice	138 (34.0)	69 (33.3)	69 (34.7)	
Wondering about fertility treatment	43 (10.6)	22 (10.6)	21 (10.6)	
Receiving fertility treatment	71 (16.4)	34 (16.4)	37 (18.6)	
Cessation of fertility treatment	16 (3.9)	10 (4.8)	6 (3.0)	
Others	7 (1.7)	7 (3.4)	0 (0.0)	
Do not want to answer	1 (0.2)	0 (0.0)	1 (0.5)	
Type of fertility treatment[1]				NS
Counseling	38	17	21	
Screening test for infertility	56	29	27	
Timed intercourse	66	34	32	
Artificial insemination	18	10	8	
In vitro fertilization	8	3	5	
Others	2	1	1	
Do not want to answer	1	0	1	
Type of medical institution for fertility treatment				NS
Specialty clinics for fertility treatment	27 (31.0)	16 (36.4)	11 (25.6)	
General gynecology clinics/hospital	53 (60.9)	23 (52.3)	30 (69.8)	
General hospital/University hospital	3 (3.4)	3 (6.8)	0 (0.0)	
Others	3 (3.4)	2 (4.5)	1 (2.3)	
Do not want to answer	1 (1.1)	0 (0.0)	1 (2.3)	
Fertility consultation for the partner				NS
Yes	37 (8.9)	26 (12.2)	11 (4.5)	
No	373 (90.1)	186 (87.3)	187 (93.0)	
Do not want to answer	4 (1.0)	1 (0.5)	3 (1.5)	

[illegible]

1. Included only a subset of the participants for whom the question applied; more than one answer may have been selected.

2. Participants whose duration of desire for pregnancy was over 10 years were assigned a value of 10 years (120 months) (3 in interventionl group and 1 in control group) .

Supplementary Table 2. Characteristics of participants who completed and did not complete the posttest

Parameters		N (%), mean ± SD		P-value
		Completed	Not completed	
Participants		659	3106	
Sex				NS
	Female	655 (99.4)	3083 (99.3)	
	Male	3 (0.5)	22 (0.7)	
	Do not want to answer	1 (0.2)	1 (0.0)	
Age (years)[1]		33.5 ± 4.9	32.4 ± 5.2	<0.0001
Overall test scores		60.9 ± 17.0	57.1 ± 16.8	<0.0001
Education				NS
	Middle high school/high school	135 (20.5)	744 (24.0)	
	Vocational school	137 (20.8)	639 (20.6)	
	National institute of technology/junior college	83 (12.6)	434 (14.0)	
	University/graduate school	294 (44.6)	1237 (39.8)	
	Do not want to answer	10 (1.5)	52 (1.7)	
Occupation				NS
	Full-time job	352 (53.4)	1715 (55.2)	
	Temporary job/Contract employee/Part-time job	151 (22.9)	734 (23.6)	
	Employer/Self-employment/Freelance	19 (2.9)	129 (4.2)	
	Not employed	122 (18.5)	445 (14.3)	
	Student	3 (0.5)	9 (0.3)	
	Others	8 (1.2)	52 (1.7)	
	Do not want to answer	4 (0.6)	22 (0.7)	
Medical/healthcare background				NS
	None	490 (74.4)	2340 (75.3)	
	Medical professional	108 (16.4)	546 (17.6)	
	Worked at medical/healthcare company	28 (4.2)	71 (2.3)	
	Studied medicine/healthcare	8 (1.2)	25 (0.8)	
	Do not want to answer	8 (1.2)	39 (1.3)	
Partner				NS
	Yes	641 (97.3)	3018 (97.2)	
	No	15 (2.3)	76 (2.4)	
	Do not want to answer	3 (0.5)	12 (0.4)	
Lives with partner				0.0066
	Yes	594 (92.7)	2661 (88.2)	
	No	47 (7.3)	352 (11.7)	
	Do not want to answer	0 (0.0)	5 (0.2)	
Annual household income				NS
	<5 million JPY	179 (27.2)	892 (28.7)	
	5-10 million JPY	308 (46.7)	1451 (46.7)	
	≥10 million JPY	68 (10.3)	365 (11.8)	
	Do not want to answer	104 (15.8)	398 (12.8)	
Prior pregnancy				NS
	Yes	229 (34.7)	1180 (38.0)	
	No	426 (64.6)	1887 (60.8)	
	Do not want to answer	4 (0.6)	39 (1.3)	
Conception method[2]				NS
	Natural	205	1073	
	Timed intercourse	16	71	
	Artificial insemination	3	20	
	In vitro fertilization	8	33	
	Others	5	16	
	Do not want to answer	1	4	
Prior history of delivery				NS
	Yes	155 (67.7)	804 (68.1)	
	No	73 (31.9)	372 (31.5)	
	Do not want to answer	1 (0.4)	4 (0.3)	
Desire for pregnancy				NS
	Yes	619 (93.9)	2886 (92.9)	
	No	32 (4.9)	184 (5.9)	
	Do not want to answer	8 (1.2)	36 (1.2)	
Duration of desire for pregnancy (months)[3]		15.8 ± 19.5	15.0 ± 21.1	NS
Action for pregnancy				0.0019
	Natural (not intended)	204 (33.0)	1211 (42.0)	
	Self-management without medical advice	202 (32.6)	764 (26.5)	
	Wondering about fertility treatment	60 (9.7)	283 (9.8)	
	Receiving fertility treatment	115 (18.6)	433 (15.0)	
	Cessation of fertility treatment	26 (4.2)	136 (4.7)	
	Others	10 (1.5)	51 (1.8)	
	Do not want to answer	2 (0.3)	8 (0.3)	
Type of fertility treatment[2]				NS
	Counseling	64	210	
	Screening test for infertility	82	305	
	Timed intercourse	104	378	
	Artificial insemination	24	118	
	In vitro fertilization	18	79	
	Others	3	8	
	Do not want to answer	1	4	
Type of medical institution for fertility treatment				NS
	Specialty clinics for fertility treatment	43 (30.5)	223 (39.2)	
	General gynecology clinics/hospital	85 (60.3)	313 (55.0)	
	General hospital/University hospital	9 (6.4)	30 (5.3)	
	Others	3 (2.1)	1 (0.2)	
	Do not want to answer	1 (0.7)	2 (0.4)	
Fertility consultation for the partner				NS
	Yes	58 (9.0)	226 (7.5)	
	No	579 (90.3)	2785 (92.3)	
	Do not want to answer	4 (0.6)	7 (0.2)	

SD; standard deviation, NS; not significant

1. Participants aged 60 or older were assigned an age of 60 (1 participant in not completed) .
2. Included only a subset of the participants for whom the question applied; more than one answer may have been selected.
3. Participants whose duration of desire for pregnancy was over 10 years were assigned a value of 10 years (120 months) (5 in completed and 51 in not completed).

Supplementary Table 3. Detailed description of questionnaire (pre- and posttest) for the assessment of fertility literacy (English translation)

Question	Contents
Question 1	What do you call the phenomenon in which a sperm and oocyte fuse?（implantation/ovulation/**fertilization**)
Question 2	Where do a sperm and oocyte find each other? (**ampulla of fallopian tube**/ uterus/ vagina/unknown)
Question 3	How long is the lifespan of an oocyte after ovulation? (1-2 hours/**12-24 hours**/2-3 days)
Question 4	How long is the lifepan of a sperm after ejaculation? (1-2 hours/12-24 hours/**2-3days**)
Question 5	Where does a fertilized egg implant in a normal pregnancy? (Fallopian tube/ovary/**uterus**)
Question 6	Which is a correct statement about the relationship between female age, menstruation, and pregnancy? (The oocyte (primordial follicle) is newly produced at each period/A woman who never menstruates is still fertile/**As you get older, it becomes harder to get pregnant**)
Question 7	What does it mean when AMH (anti-Müllerian hormone) is low?（**The number of primordial follicle is low**/Low sperm count/Thin endometrium）
Question 8	When is the best time to get pregnant during your menstrual cycle? (during menstruation/**the day before ovulation**/when the next period comes)
Question 9	What is the proper location to measure basal body temperature? (ear hole/armpit/**under the tongue**)
Question 10	Which of the following should not be taken by both women and men when aiming for pregnancy? (**tobacco/**alcohol/caffeine)
Question 11	Which is a correct statement about your lifestyle and pregnancy? (Having a healthy lifestyle makes you fertile/**Gaining too much weight makes it harder to get pregnant**/It is easier to get pregnant if you are more skinny than your standard body type)
Question 12	What is the proportion of couples with infertility problems in Japan? (1 in 2-3 couples/ **1 in 5-6 couples**/ 1 in 9-10 couples)
Question 13	Which is an appropriate duration for suspecting infertility issues if you have not yet gotten pregnant in the absence of contraception? (3 months/**1 year**/2 years)
Question 14	Of those experiencing infertility, what percentage are due to issues related to the man? (10-20%/**40-50%**/70-80%)
Question 15	Which is the most common cause of male infertility? (**problem with sperm (disorders of spermatogenesis)**/ trouble in sexual intercourse (sexual dysfunction)/problem with the passage of sperm (ejaculatory duct obstruction))
Question 16	Which is covered by public insurance in principle? (**timed intercourse**/ artificial insemination/ *in vitro* fertilization)
Question 17	Where would you apply to request access to the public financial subsidy system for fertility treatment? (workplace/**local government**/Ministry of Health, Labor and Welfare)
Question 18	Which blood test suspects the woman to have a low number of oocytes? (**high FSH and low AMH**/ high prolactin/high LH and AMH)
Question 19	Which of the following cannot be detected by a routine ultrasonographic test? (endometrial thickness/timing of estimated ovulation/**fallopian tube patency**)
Question 20	Which of the following cannot be detected by hysterosalpingography? (fallopian tube patency/the shape of intrauterine cavity/**the number and size of uterine fibroids**)
Question 21	What does Huhner test examine? (**whether sperm reached the uterus**/whether the number of eggs is small/whether a sperm and oocyte fertilized)
Question 22	What kind of test is semen analysis? (to collect blood and count the number of sperm in the blood/**to collect semen by masturbation and check them under a microscope**/to apply ultrasonography to testes and check them)
Question 23	When is the best time to have sexual intercourse when aiming for pregnancy with the timed intercourse method? (two days after the ovulation test showed strongly positive/**the day when the follicle became 18-20 mm by ultrasonography**/three days after basal body temperature has been in high-temperature phase)
Question 24	What kind of treatment is artificial insemination? (to inject a sperm into the retrieved oocyte/to mechanically inject sperm into the ovaries/**to inject sperm into the uterus**)
Question 25	What is the pregnancy rate for artificial insemination? (**3-10%**/20-30%/50-60%)
Question 26	Which is a correct treatment flow for *in vitro* fertilization? (**ovarian stimulation → oocyte retrieval → fertilization → embryo transfer**/oocyte retrieval → ovarian stimulation → fertilization → embryo transfer/ovarian stimulation → embryo transfer → oocyte retrieval → fertilization)
Question 27	After *in vitro* fertilization, how long do you usually culture the fertilized eggs *in vitro*? (**2-5 days**/7-10 days/10-14 days)
Question 28	What is the pregnancy rate for *in vitro* fertilization? (3-10%/**20-30%**/50-60%)

Participants were asked to select one of three choices or "I do not know". Bold and underlined option was recognized as the correct answer.
FSH, follicle stimulating hormone; LH, luteinizing hormone

Supplementary Table 4. Summary of provided information during the 1-week provision

Days	Control group (Women's healthcare and general gynecology)	Contents		Intervention group (Fertility and fertility treatment)	Contents	
		Words	Figures		Words	Figures
Day 1	Normal menstruation<br>-cycle, duration, amount, symptoms	1081	0	Mechanism of prengnancy<br>Relationship between age and fertility	1808	6
Day 2	Normal vaginal discharge	1054	0	How to find the best timing for getting pregnant<br>Basal body temperature and other self-healthcare management	3590	9
Day 3	Life stage and hormone	527	1	Pre-conception care<br>Infertility	3664	9
Day 4	Postmenopausal disorder-physical and psychological symptoms	1431	0	Initial assessment in fertility treatment	2070	2
Day 5	Gynecological disorder and sexually transmitted disease<br>-disease associated with dysmenorrhea and abnormal discharge	899	1	Examination in fertility treatment<br>blood test, ultrasonography, hysterosalpingography,<br>Huhner test, semen analysis	3721	7
Day 6	Gynecological examination	905	2	Methods of fertility treatment<br>-timed intercourse, artificial insemination, *in vitro* fertilization	4451	5
Day 7	Symptoms during pregnancy<br>Symptoms after delivery (postpartum health care)	1873	1	Male infertility<br>Expense of fertility treatment	2635	2
