# Supplementary material for: Smartphone application improves fertility treatment-related literacy in a large-scale virtual randomized controlled trial in Japan
Source: NPJ Digit Med. 2021 Nov 30;4:163. doi: 10.1038/s41746-021-00530-4 (PMC8632894; doi:10.1038/s41746-021-00530-4)
Supplement: Supplementary file 2 — Reporting Summary [file 41746_2021_530_MOESM2_ESM.pdf]

nature portfolio

Corresponding author(s): Akihiro Umezawa

Last updated by author(s): Oct 16, 2021

Reporting Summary

Nature Portfolio wishes to improve the reproducibility of the work that we publish. This form provides structure for consistency and transparency in reporting. For further information on Nature Portfolio policies, see our Editorial Policies and the Editorial Policy Checklist.

Statistics

For all statistical analyses, confirm that the following items are present in the figure legend, table legend, main text, or Methods section.

n/a	Confirmed	
☐	☒	The exact sample size (*n*) for each experimental group/condition, given as a discrete number and unit of measurement
☒	☐	A statement on whether measurements were taken from distinct samples or whether the same sample was measured repeatedly
☐	☒	The statistical test(s) used AND whether they are one- or two-sided<br>*Only common tests should be described solely by name; describe more complex techniques in the Methods section.*
☒	☐	A description of all covariates tested
☒	☐	A description of any assumptions or corrections, such as tests of normality and adjustment for multiple comparisons
☒	☐	A full description of the statistical parameters including central tendency (e.g. means) or other basic estimates (e.g. regression coefficient) AND variation (e.g. standard deviation) or associated estimates of uncertainty (e.g. confidence intervals)
☐	☒	For null hypothesis testing, the test statistic (e.g. *F*, *t*, *r*) with confidence intervals, effect sizes, degrees of freedom and *P* value noted<br>*Give P values as exact values whenever suitable.*
☒	☐	For Bayesian analysis, information on the choice of priors and Markov chain Monte Carlo settings
☒	☐	For hierarchical and complex designs, identification of the appropriate level for tests and full reporting of outcomes
☒	☐	Estimates of effect sizes (e.g. Cohen's *d*, Pearson's *r*), indicating how they were calculated

Our web collection on statistics for biologists contains articles on many of the points above.

Software and code

Policy information about availability of computer code

Data collection	Through a proprietary smart phone application called Luna Luna (MTI, Ltd.) downloadable for free
Data analysis	SPSS (IBM) and Prism 8.01 software (GraphPad, Inc.) were used for the statistical analyses.

For manuscripts utilizing custom algorithms or software that are central to the research but not yet described in published literature, software must be made available to editors and reviewers. We strongly encourage code deposition in a community repository (e.g. GitHub). See the Nature Portfolio guidelines for submitting code & software for further information.

Data

Policy information about availability of data

All manuscripts must include a data availability statement. This statement should provide the following information, where applicable:
- Accession codes, unique identifiers, or web links for publicly available datasets
- A description of any restrictions on data availability
- For clinical datasets or third party data, please ensure that the statement adheres to our policy

The datasets generated during and/or analyzed during the current study are available from the corresponding author on reasonable request.

Field-specific reporting

Please select the one below that is the best fit for your research. If you are not sure, read the appropriate sections before making your selection.

☐ Life sciences ☒ Behavioural & social sciences ☐ Ecological, evolutionary & environmental sciences

For a reference copy of the document with all sections, see nature.com/documents/nr-reporting-summary-flat.pdf

Behavioural & social sciences study design

All studies must disclose on these points even when the disclosure is negative.

Study description	Quantitative experimental study
Research sample	A total of 4,137 Luna Luna users agreed to participate and were administered the questionnaire and pretest.
Sampling strategy	Users were requested to participate in this study by sending Luna Luna in-application notifications about the outline of the research plan and how data collected from the application would be used. Furthermore, MTI Ltd. published a press release about the study, together with information on how to voluntarily participate. We recruited participants between June 18 and 25, 2020. The recruitment ended on June 25 because the number of participants reached to the pre-estimated number. Sample size calculations were performed based on detecting a difference of 20% in score between two groups and setting the type I and II error rate at 0.05 and 0.1, respectively. Mean score of in the control group was expected to be about 50%, resulting in a total number of 250 individuals (at a 1:1 scheme resulting in 125 per group). Considering an expectedly high withdrawal rate at follow-up for this application-based study, we empirically assumed that a baseline population of nearly 4,000 participants allocated to the group would result in sufficient numbers based on the response and withdrawal rates experienced in previous surveys conducting through Luna Luna, MTI Ltd.
Data collection	A smart phone application called "Luna Luna" was used to collect baseline data as well as pretest and posttest responses and questionnaire.
Timing	We recruited participants between June 18 and 25, 2020, and performed a 4 week follow-up on all participants.
Data exclusions	We excluded duplicate users of Luna Luna and Taion note.
Non-participation	A total of 4,137 Luna Luna users agreed to participate and were administered the questionnaire and pretest, among which 3,765 participants (91.0 %) responded and were randomly allocated into either the intervention group (N=1,883) or the control group (N=1,882).
Randomization	We performed computer-based stratified randomization which involved separating the participants into 29 strata based on the performance on the pretest, i.e., based on the number of correct answer (0 to 28) in order to allocate the participants into two groups for equally distribution of distribute high to low performance, assigning a unique randomly generated number to each participant within each strata, and alternating allocation of participants sequentially to intervention or control group for each strata.

Reporting for specific materials, systems and methods

We require information from authors about some types of materials, experimental systems and methods used in many studies. Here, indicate whether each material, system or method listed is relevant to your study. If you are not sure if a list item applies to your research, read the appropriate section before selecting a response.

Materials & experimental systems

n/a	Involved in the study	
☒	☐	Antibodies
☒	☐	Eukaryotic cell lines
☒	☐	Palaeontology and archaeology
☒	☐	Animals and other organisms
☐	☒	Human research participants
☐	☒	Clinical data
☒	☐	Dual use research of concern

Methods

n/a	Involved in the study	
☒	☐	ChIP-seq
☒	☐	Flow cytometry
☒	☐	MRI-based neuroimaging

Human research participants

Policy information about studies involving human research participants

Population characteristics	User of the reproductive health smart phone application Luna Luna comprising predominately women (99%), whose mean age (32.6 years) appeared similar to the characteristics of the general Japanese population.

Recruitment	"Luna Luna" application software users who were interested in this health literacy study were recruited through in-app notifications and public advertisement.
Ethics oversight	National Center for Child Health and Development

Note that full information on the approval of the study protocol must also be provided in the manuscript.

Clinical data

Policy information about clinical studies

All manuscripts should comply with the ICMJE guidelines for publication of clinical research and a completed CONSORT checklist must be included with all submissions.

Clinical trial registration	UMIN000040721
Study protocol	We have submitted the trial protocol in this submission.
Data collection	Using smart phone application software "Luna Luna", between June 18 and 25, 2020.
Outcomes	Improvement in infertility-related literacy defined as the difference in score between the pretest and posttest.
